# Physicians’ Study Travels

**Published:** 1913-11

**Authors:** R. M. Harbin

**Affiliations:** Secretary; Rome, Ga.


### PHYSICIANS' STUDY TRAVELS.

Rome, Ga., October 24, 1913.

Editor Journal-Record of Medicine:

I enclose a copy of a letter which may be of interest to you. I have reviewed the by-laws of the American Society for Physicians' Study Travels, and in substance all the executive powers of the club are vested in the Executive Committee, and this seems necessary to facilitate the workings of the club.

I would suggest from my investigation that:

1. The Executive Committee and those of the Committee on Arrangements who are interested, work out a brief form of by-laws to be enacted.

2. The only qualification for membership being membership in the State Association (other states as well).

3. Terms of officers be made long for obvious reasons.

4. All the details, place of meeting, program, etc., to be entirely left to the Executive Committee.

5. Assessments of dues will only be made on those who attend the meetings.

It seems expedient to the secretary to have the form of by-laws printed and mailed to those who have asked to be enrolled as members.

To the committeemen I would suggest mailing the programs to the entire membership of the Association (and outside the state as well), and stamp on programs "Notify Dr. W. S. Goldsmith if you can attend, and the Secretary-Treasurer if you wish to be kept on the mailing list. Your attending once does not compel membership."

I have sent you a copy of Dr. R. Matas' invitation to the New Orleans clinics. While February 27 and 28th is early in the year, the reduced rates, pleasant season and the opportunity to attend Mardi Gras make reasons for accepting the invitation. Mail any suggestions you can offer to Dr. W. S. Goldsmith.

Yours truly,

R. M. HARBIN,

Secretary.

